# Proanthocyanidins from Ginkgo extract EGb 761^®^ improve bioenergetics and stimulate neurite outgrowth *in vitro*

**DOI:** 10.3389/fphar.2025.1495997

**Published:** 2025-06-12

**Authors:** Imane Lejri, Ina Vukalović, Amandine Grimm, Anne Eckert

**Affiliations:** ^1^ Neurobiology Lab for Brain Aging and Mental Health, University Psychiatric Clinics UPK, Basel, Switzerland; ^2^ Research Cluster Molecular and Cognitive Neurosciences, University of Basel, Basel, Switzerland; ^3^ Department of Biomedicine, University of Basel, Basel, Switzerland

**Keywords:** EGb 761^®^, proanthocyanidins, mitochondria, bioenergetics, neurite outgrowth

## Abstract

EGb 761^®^ is a proprietary extract from *Ginkgo biloba* leaves approved as an herbal medication for the treatment of dementia and its related disorders. Preclinical studies highlight antioxidant, ROS scavenging, mitochondria-stabilizing, and neuroplastic properties as some of the reported pharmacological activities. Efficacy is traditionally ascribed to terpene lactones and flavone glycosides. However, these quantified known active compounds in EGb 761^®^ only cover approximately 30% of the mass balance, and there is the possibility that additional compounds from the residual 70% may enhance the activity of the quantified extract EGb 761^®^. Proanthocyanidins (PACs) are a quantitatively relevant component in EGb 761^®^, and some pharmacological activity has been reported for PACs from Ginkgo and other herbal sources. In this study, we focused on the effects of EGb 761^®^ and its isolated PACs on mitochondrial bioenergetics and neuroplasticity in the human neuroblastoma cell line SH-SY5Y. We successfully demonstrated positive effects of EGb 761^®^ and its isolated PACs on several mitochondrial characteristics and neurite outgrowth. As PACs exhibited similar effects compared to the respective extract concentration, they can be considered a pharmacologically relevant component of EGb 761^®^.

## Introduction

EGb 761^®^ is a special extract produced from dried *Ginkgo biloba* leaves (*Ginkgo biloba* L. [*Ginkgoaceae*]). Medicinal products containing EGb 761^®^ as the active substance have shown efficacy in patients with dementia and cognitive impairment (Gauthier and Schlaefke, 2014; Tan et al., 2014; von Gunten et al., 2016; Savaskan et al., 2018; Müller et al., 2019; Riepe et al., 2025), tinnitus (von Boetticher, 2011), and vertigo (Hamann, 2007). The composition of EGb 761^®^ is adjusted to the specifications of the European Pharmacopeia (European Medicines Agency, 2015). Pharmacologically active ingredients are enriched during the extraction process. Primary extraction is carried out with aqueous acetone (60%, w/w), and the extract is modified to 22%–27% ginkgo flavonoids; 5.4%–6.6% terpene trilactones, consisting of 2.8%–3.4% ginkgolides A, B, and C (Supplementary Figure S1); and 2.6%–3.2% bilobalide (Supplementary Figure S1) (calculated on the dried extract) (Eckert, 2012; Ihl, 2013). A range of different modes of action have been reported for other Ginkgo extracts and EGb 761^®^. These encompass an increase in neurotransmitter signaling (Kehr et al., 2012), reduction of amyloid-β plaque deposition in transgenic mouse models (Qin et al., 2018; Nguyen et al., 2025), attenuation of neuroinflammation (Unger, 2013; Gargouri et al., 2018), stimulation of neuroplasticity, and improved mitochondrial function (Eckert et al., 2003; Zhang et al., 2022), among others.

One mechanism that potentially contributes to some of the observed effects could be the antioxidant properties and the scavenging of reactive oxygen species (ROS) (Abdel-Kader et al., 2007; Ude et al., 2013), which, by protecting the electron transport chain (ETC) complexes from oxidative stress, leads to improved mitochondrial respiration and increased ATP availability (Abdel-Kader et al., 2007; Hoerr et al., 2022). The free radical scavenging activity of Ginkgo constituents of EGb 761^®^ was demonstrated in several preclinical models (Smith and Luo, 2003; Qa’dan et al., 2011; Ude et al., 2013; Sens-Albert et al., 2021; Li et al., 2021; Anaya-Fernández et al., 2024), showing a reduction in free radical accumulation, oxidative damage, and apoptosis.

Preclinical studies suggest that terpene trilactones and Ginkgo flavonoids mediate some of the pharmacological effects of Ginkgo extracts but cannot account for the overall activity profile alone. In regulatory terms, the EU specifies herbal medicinal products containing Ginkgo extracts as the so-called quantified extracts with specified amounts of terpene trilactones and flavonoids that together cover approximately 30% of the mass balance. The main flavonol constituents are quercetin and kaempferol, typically present as O-glycosides (Supplementary Figure S2). However, whether constituents from the remaining 70% of the mass balance also contribute to the overall activity is currently not established.

Using a newly developed quantitative HPLC method analysis, it has been recently shown that EGb 761^®^ contains an average concentration of 7% of proanthocyanidins (PACs) (Supplementary Figure S3) (Kulić Z. et al., 2022). Due to the precise and standardized process of extract preparation for EGb 761^®^, batch-to-batch consistency is high (Kulić et al., 2022b). So far, some available research studies showed evidence of the antioxidant and radical scavenging effect of Ginkgo PACs *in vitro* (Qa’dan et al., 2011) and neuroprotective effects in an ischemia-reperfusion model *in vivo* (Yao et al., 2020). Additionally, PACs from Ginkgo and other sources exerted activity against amyloid-beta aggregation and cognitive deficiency in AD transgenic mouse models (Wang et al., 2008; Xie et al., 2014). PACs might contribute to the pharmacological profile of EGb 761^®^ as a quantitatively relevant and pharmacologically active compound (Sens-Albert et al., 2021; Kulić Z. et al., 2022; Kulić et al., 2022b). Nevertheless, more insights are needed on the precise mechanisms of the effects of PACs on a cellular level.

In this study, we aimed to assess the prospective contribution of PACs to the pharmacological activity of the Ginkgo extract EGb 761^®^. To this aim, we conducted a comparative *in vitro* analysis of the effects of an isolated PAC fraction compared to the unfractionated extract on mitochondrial bioenergetics and neuroplasticity in the human SH-SY5Y neuroblastoma cell line.

## Materials and methods

### Chemicals and reagents

The chemicals and reagents used included Dulbecco’s modified Eagle medium (DMEM) (Invitrogen), phosphate-buffered saline (PBS) (Dominique Dutscher), fetal bovine serum (FBS) (Corning), horse serum (HS) (BioConcept), penicillin/streptomycin (BioConcept), GlutaMAX (Thermo Fisher Scientific), Accutase (Innovative), dimethylsulfoxide (DMSO) (Sigma-Aldrich), 3-(4,5-dimethylthiazol-2-yl)-2,5-diphenyltetrazolium bromide (MTT) (Sigma-Aldrich), ATPlite 1step Luminescence Assay Kit (Perkin Elmer), trypan blue (Thermo Fisher Scientific), Hank’s balanced salt solution (HBSS) (Sigma-Aldrich), tetramethylrhodamine-methylester (TMRM) (Sigma Aldrich), MitoSOX (Invitrogen), D-glucose (Sigma-Aldrich), pyruvate (Sigma-Aldrich), L-glutamine (Sigma-Aldrich), CellTracker Blue CMAC (Invitrogen), gelatin (Merck), paraformaldehyde (PFA) (Sigma-Aldrich), oligomycin (Sigma-Aldrich), rotenone (Sigma-Aldrich), antimycin (Sigma-Aldrich), XF Calibrant Seahorse (Agilent Technologies), XF DMEM Seahorse (Agilent Technologies), MitoTracker Red CMXROS (Invitrogen), MitoTracker Green FM (Invitrogen), RNeasy Mini Kit (25) (Qiagen), GoScript™ Reverse Transcription Mix, Oligo (dT) (Promega), GoTaq^®^ Probe qPCR Master Mix (Promega), primers for RT-qPCR (Microsynth AG), neurobasal medium NB (Sigma-Aldrich), retinoic acid RA (Sigma-Aldrich), B27 (Gibco Invitrogen), nerve growth factor (NGF) (Lubio), rat tail collagen I (Corning), anti-βIII-tubuline (Sigma-Aldrich), and Alexa Fluor 488-conjugated secondary antibody (Abcam).

*Ginkgo biloba* extract EGb 761^®^ (batch PSC0148/WS1133/EXCh.053) was produced and supplied by Dr. Willmar Schwabe GmbH & Co. KG, Karlsruhe, Germany. The PACs fraction (batch PSC0148/WS1133/EXCh.053/B/Rö16-235-C) was isolated from EGb 761^®^ by multiple column chromatography, as described in the literature (Kulić Z. et al., 2022), and has a content of 90.81% Ginkgo PACs and 9.19% residual solvent (water).

### Cell culture

Human SH-SY5Y neuroblastoma cells were maintained at 37°C in a humidified incubator with 5% CO_2_ in DMEM supplemented with 10% (v/v) heat-inactivated FCS, 5% horse serum, and 1% penicillin–streptomycin–GlutaMAX (PSG). Cells were passaged biweekly and plated 1 day prior to treatment, when 80% confluency was reached.

For experiments, cells were plated with 6–12 replicates into clear-bottom 96-well cell culture plates at a density of 1 × 10^4^ cells/well, unless otherwise specified.

### Coating

For differentiation, 96-well plates were coated with collagen type I (rat tail BD Bioscience) at 0.05 mg/mL for 3 h, followed by cell seeding.

Seahorse XF Cell Culture miniplates were coated with 0.1% gelatin for 30 min.

No coating was applied for other experimental conditions.

### Treatment paradigm

The selection of doses was based on the literature, where common concentrations of EGb 761^®^ or other Ginkgo extracts are typically reported to range between 1 and 100 μg/mL (Lejri et al., 2019; Rhein et al., 2010). EGb 761^®^ contains approximately 7% PACs. To compare the effects of PACs to the corresponding concentrations of EGb 761^®^, PACs concentrations were selected accordingly. EGb 761^®^ (1, 10, and 100 μg/mL) or PACs (0.1, 1, and 10 μg/mL) treatments were applied 24 h post-seeding. Treatment duration was 24 h. Control specimens received 10 µL/well DMEM supplemented with 1% PSG. DMSO 0.1% was used as the vehicle control.

Treatment solutions were prepared from the dried extract and isolated fraction in DMSO and serum-free DMEM supplemented with 1% PSG.

### Differentiation protocol for microscopy and neurite outgrowth analysis

SH-SY5Y neuroblastoma cells were seeded into black 96-well cell culture plates at 5 × 10^3^ cells/well in high-glucose DMEM (+10% FCS and 1% PSG).

Differentiation was induced with neurobasal medium, containing 1% PSG, 2% B27, and retinoic acid (RA, 10 µM), which was added for 3 days (Lejri et al., 2019). Post-treatment with EGb 761^®^ and PACs, cells were fixated with 2% paraformaldehyde. DMSO 0.1% and NGF (50 ng/mL) served as negative and positive controls, respectively. Media were refreshed every second day.

### CellTracker Blue dye loading

To account for differences in cell number, readouts were normalized to cell count using CellTracker Blue staining. CellTracker Blue CMAC (7-amino-4-chloromethylcoumarin) was loaded at 5 µM and incubated for 1 h at 37°C at 5% CO_2_ (Szabo et al., 2024). CMAC was co-incubated with other dyes, following the respective protocols specific to each dye during combined staining procedures. Fluorescence was measured at 353 nm (excitation)/466 nm (emission) using the Cytation 3 reader.

### Determination of superoxide anion levels

Two days prior to the assay, the cells were plated with 6–12 replicates into a black 96-well cell culture plate at a density of 10,000 cells per well. The following day, the treatment protocol was followed. On the day of the assay, the cells were loaded with a final concentration of 5 µM of MitoSOX and then incubated for 2 h at 37°C and 5% CO_2_. After washing twice with HBSS, fluorescence was detected using the Cytation 3 cell imaging multi-mode reader at 531 nm (excitation)/595 nm (emission) (Szabo et al., 2024).

### MMP assay

Mitochondrial membrane potential (MMP) was assessed using TMRM at 0.4 μM for 30 min in the dark (Szabo et al., 2024). The cells were plated with 6–12 replicates into a black 96-well cell culture plate at a density of 10,000 cells per well. The following day, the treatment protocol was followed. Two days after plating, the cells were treated with the dye at a final concentration of 0.4 µM, and afterward they were incubated for 30 min under agitation at room temperature. Before measuring the fluorescence at 531 nm (excitation)/595 nm (emission) using the Cytation 3 cell imaging multi-mode reader (BioTek), the cells were washed twice with HBSS.

### ATP levels

The total ATP was measured using the ATPlite 1step bioluminescence kit (Perkin Elmer) as per the manufacturer’s protocol, with luminescence detected by the multiplate reader Cytation 3 (BioTek). Two days prior to the assay, the cells were plated with 6–12 replicates into a black 96-well cell culture plate at a density of 10,000 cells per well. The following day, the treatment protocol was followed. On the day of the assay, after the preparation for the ATP standard curve, 100 µL of the ATP substrate solution was added to each well. After incubation in the dark under agitation at room temperature for 5 min, luminescence was measured using the Cytation 3 cell imaging multi-mode plate reader (BioTek) (Szabo et al., 2024).

### Metabolic activity assay

Forty-eight hours prior to the assay, the cells were plated with 6–12 replicates into a 96-well cell culture plate at a density of 10,000 cells per well. The following day, the treatment protocol was followed. On the day of the assay, 10 µL of MTT solution (5 mg/mL MTT (3-(4,5-dimethylthyazol-2-yl)-2,5-diphenyltetrazolium bromide)) were added to every well and incubated for 3 h at 37°C and 5% CO_2_. During the incubation time, metabolically active cells transformed MTT to formazan crystals. After adding DMSO to dissolve the formazan crystals, the absorbance was measured at 550 nm using the Cytation 3 cell imaging multi-mode plate reader (BioTek).

### Mitochondrial mass

Cells were plated with 6–12 replicates into a black 96-well cell culture plate at a density of 10,000 cells per well 48 h before the assay. The following day, the treatment protocol was followed. On the day of the assay, the cells were incubated for 1 h at 37°C and 5% CO_2_ with MitoTracker Green FM (100 nM) and then washed with HBSS. Fluorescence was detected using the Cytation 3 cell imaging multi-mode reader (BioTek) at 490 nm (excitation)/516 nm (emission).

### RNA isolation, cDNA synthesis, and qRT-PCR

SH-SY5Y cells were plated into 6-well cell culture plates at 10^5^ cells/well (Szabo et al., 2023). EGb 761^®^ (10 μg/mL) and PACs (1 μg/mL) treatment duration was 24 h. After PBS washing and cell lysis, total RNA was isolated using the RNeasy Mini Kit. RNA concentration and purity were quantified and assessed using NanoDrop. RNA was reverse transcribed to cDNA using the GoScript™ Reverse Transcription Mix, Oligo (dT). qRT-PCR was performed using the GoTaq^®^ qPCR Master Mix (ref. dye CXR, 300 nM/primers 900 nM) with the StepOnePlus^TM^ Real-Time PCR System. The relative fold gene expression was quantified using the 2^−ΔΔCT^ method with GAPDH as an endogenous reference.

### Profiling mitochondrial respiration

The Seahorse XF HS Mini Analyzer (Agilent Technologies) was used to measure the oxygen consumption rate (OCR) and extracellular acidification rate (ECAR), with SH-SY5Y cells plated at 1.5 × 10^4^ cells/well (Szabo et al., 2024). The XF Mito Stress Test protocol was followed as per the manufacturer’s specifications. Mitochondrial agents were injected at the following concentrations: oligomycin (2 μM) and a combination of antimycin A (1 μM) and rotenone (1 μM). The assay medium consisted of the Seahorse XF DMEM (pH of 7.4) with additional 18 mM D-glucose, 4 mm pyruvate, and 2 mM L-glutamine.

### Immunostaining

Cell nuclei were stained with DAPI (3 μM, 10 min of incubation), and neurites were immunolabeled with anti-βIII-tubulin (5 μg/mL, incubation overnight) and Alexa Fluor 488-conjugated secondary antibody (5 μg/mL, 1 h of incubation) (Lejri et al., 2019).

### Microscopy and analysis (software)

Images were captured using the Cytation 3 cell imaging multi-mode reader (×20 objective) and analyzed with ImageJ (neurophology plugin). The following parameters of neuroplasticity were evaluated: the number of contact and branching points (endpoint and attachment point), total neurite length, soma count, and neurite count. In total, approximately 10,000 cells and 57,000–101,000 neurites were evaluated. Images were analyzed to quantify neurite extension between cells, which were visualized using anti-βIII-tubulin/Alexa488 staining for neurites and DAPI for nuclei.

### Statistical analysis

Data are presented as the mean ± SEM, normalized first to the cell count with CellTracker Blue and afterward to the control condition (CTRL = 100%). Statistical analysis was performed using the GraphPad Prism software. When comparing more than two experimental groups, one-way ANOVA and *post hoc* Dunnett’s multiple comparisons test versus control were applied. For direct comparisons between two groups, an unpaired Student’s *t*-test was used. *P*-values <0.05 were considered statistically significant.

## Results

To assess the effects of EGb 761^®^ and PACs, the human neuroblastoma cell line SH-SY5Y received treatments of EGb 761^®^ at concentrations of 1/10/100 μg/mL or PACs at the roughly corresponding concentrations of 0.1/1/10 μg/mL for 24 h (reflecting PACs 7% content in EGb 761^®^). A potential toxic effect of the vehicle control 0.1% DMSO (final assay concentration) was ruled out in prescreening tests, such as the ATP and MTT assays indicating metabolic activity/cell viability, and is, therefore, not shown in the following assay results.

### Antioxidant effects of EGb 761^®^ and PACs on MMP and ATP production

**FIGURE 1:**
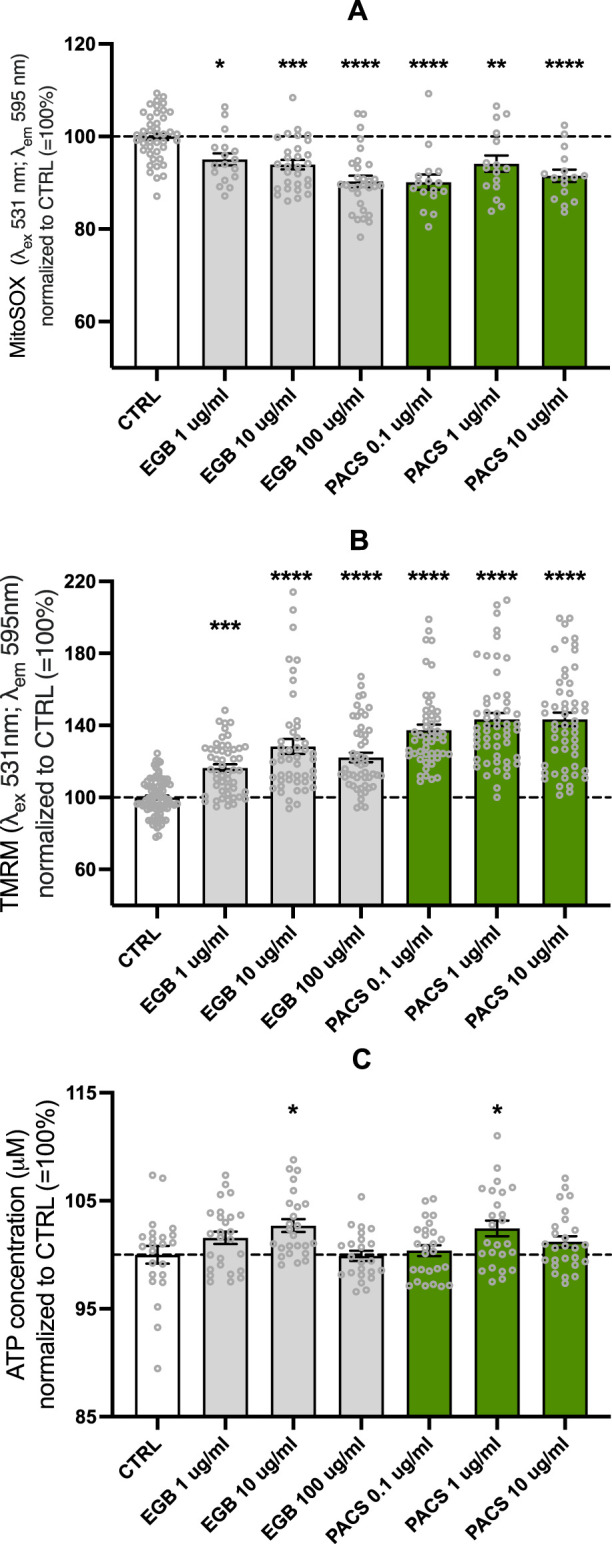
EGb 761^®^ and PACs decreased mitochondrial superoxide **(A)** and increased mitochondrial membrane potential **(B)** and ATP levels **(C)**. Values represent the mean ± SEM of three independent experiments for **(A)** and five independent experiments for **(B, C)**. Each open circle represents one replicate. Values were normalized to 100% of untreated CTRL cells. Statistical analysis was performed with ANOVA and *post hoc* Dunnett’s multiple comparisons test. *P < 0.05, **p < 0.01, ***p < 0.001, and ****p < 0.001 compared to control condition. CTRL, control; PACs, proanthocyanidins-fraction; EGb, EGb 761^®^ extract.

Both EGb 761^®^ and PACs exhibited antioxidant and ROS scavenging properties as treatment with both compounds led to a decrease in mitochondrial superoxide in the neuroblastoma cell line SH-SY5Y. Lowest concentrations of mitochondrial superoxide were measured in the treatment group of EGb 761^®^ 100 μg/mL (−9.7% compared to CTRL, *p* < 0.0001) and PAC 0.1 μg/mL (−9.9% compared to CTRL, *p* < 0.0001) conditions (Figure 1A). ROS have shown direct effects on the electron transport chain, OXPHOS, and ATP production. Therefore, we decided to investigate the MMP and total ATP levels in the following assays. EGb 761^®^ (10 μg/mL, *p* = 0.0179) and PAC (1 μg/mL, *p* = 0.0416) concentrations positively impacted the total ATP production in neuroblastoma cells (Figure 1C). Other concentrations did not alter total ATP levels compared to the control. To further validate the beneficial effects of both compounds on ATP production, measuring the MMP was crucial, as it plays a critical role in driving oxidative phosphorylation. Both PACs and EGb 761^®^ increased MMP, but PACs showed a more pronounced effect than EGb 761^®^. Specifically, PACs treatment elevated the mitochondrial membrane potential up to 42% in comparison to the control condition, with both 1 μg/mL and 10 μg/mL exhibiting similar results (for both *p* < 0.0001), suggesting a ceiling effect. In contrast, treatment with EGb 761^®^ revealed its maximal effect at a concentration of 10 μg/mL (*p* < 0.0001), resulting in a 30% increase. The highest concentration led to only a 22% elevation (*p* < 0.0001), which is slightly above the 16% (*p* < 0.0001) observed at the lowest concentration (Figure 1B).

### Enhanced metabolic activity, mitochondrial mass, and biogenesis by EGb 761^®^ and PACs

**FIGURE 2:**
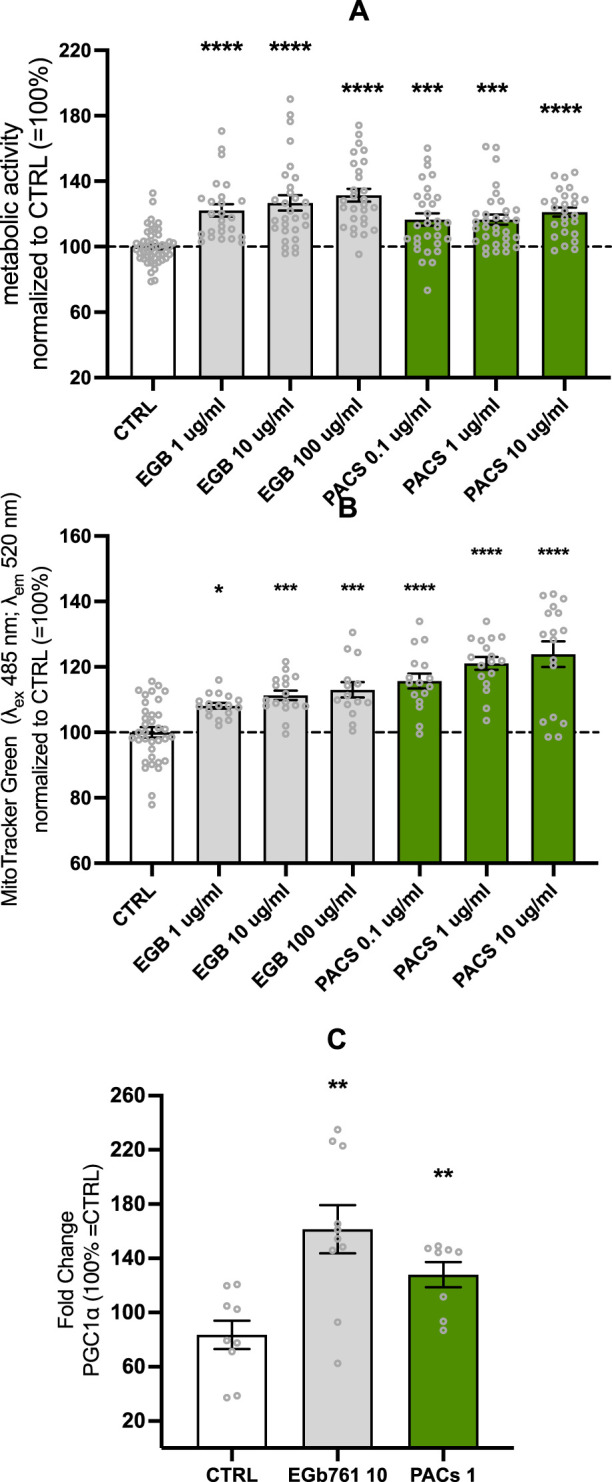
EGb 761^®^ and PACs increase metabolic activity **(A)** and mitochondrial **(B)** mass, while modulating the expression of PGC1-α **(C)** in SH-SY5Y cells. Optimal effects on metabolic activity and mitochondrial mass were achieved under the conditions of EGb 761^®^ at 10 μg/mL and PACs at 1 μg/mL. Consequently, gene expression was evaluated under these respective conditions. Values represent the mean ± SEM of five independent experiments for **(A)** and three independent experiments for **(B, C)**. Each open circle represents one replicate. Values were normalized to 100% of untreated CTRL cells. Statistical analysis was performed with ANOVA and *post hoc* Dunnett’s multiple comparisons test. *P < 0.05, **p < 0.01, ***p < 0.001, and ****p < 0.001 compared to the control condition. CTRL, control; PACs, proanthocyanidins-fraction; EGb, EGb 761^®^ extract.

Increases in ATP and MMP were paralleled by elevated metabolic activity. The metabolic activity was significantly increased up to 31% for the EGb 761^®^ 100 μg/mL-treated cells (*p* < 0.0001) and up to 21% for the PAC 10 μg/mL-treated cells (*p* < 0.0001) (Figure 2A). Lower concentrations also demonstrated substantial effects on EGb 761^®^ at 10 and 1 μg/mL of EGb 761^®^ increased activity by 26% and 22%, respectively (both *p* < 0.0001), whereas PACs at 1 and 0.1 μg/mL resulted in a 16% increase, with both concentrations to 16% (*p* = 0.0006 and *p* = 0.0009, respectively). Ameliorated metabolic activity and ATP levels come with increased energy demands; therefore, investigating effects on mitochondrial mass was the next logical step.

The increase in mitochondrial mass in human neuroblastoma cells seemed to be cohesive with the results from previous experiments. PACs had a slightly better effect on mitochondrial mass than EGb 761^®^, with mitochondrial mass increases of 15%, 21%, and 24% at 0.1, 1, and 10 μg/mL (all *p* < 0.0001), respectively. EGb 761^®^ treatment led to elevations of 8% (*p* = 0.0266), 11% (*p* = 0.0005), and 13% (*p* = 0.0002) at 1, 10, and 100 μg/mL, respectively (Figure 2B).

PPARGC1-α is coding for a transcriptional coactivator regulating gene involved in energy metabolism, linking external stimuli and the regulation of mitochondrial biogenesis. Based on the optimal conditions for metabolic activity and mitochondrial mass—EGb 761^®^ at 10 μg/mL and PACs at 1 μg/mL—gene expression was assessed accordingly. EGb 761^®^ and PAC treatment of the SH-SY5Y cell line increased the protein expression of PPARGC1-α after 24 h (Figure 2C), suggesting upregulated mitochondrial biogenesis. EGb 761^®^ at 10 μg/mL elevated PPARGC1-α expression by 60% (*p* = 0.0019), whereas PACs only elevated it by 27% (*p* = 0.0068).

### Bioenergetic enhancements and metabolic shift

**FIGURE 3:**
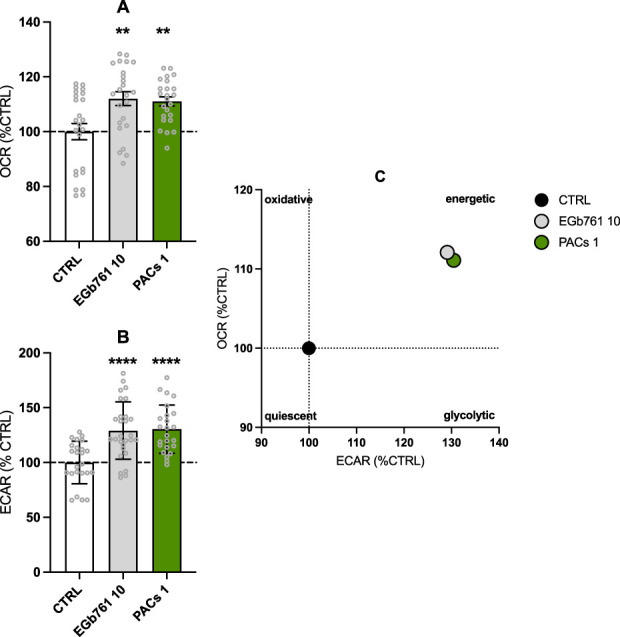
EGb 761^®^ and PACs increase the oxygen consumption rate (OCR) **(A)** and extracellular acidification rate (ECAR) **(B)** in human neuroblastoma cells. Treatment with PACs at 1 μg/mL and EGb 761^®^ at 10 μg/mL led to a more energetic state **(C)**. Values represent the mean ± SEM of three independent experiments and six replicates per condition. Each replicate is measured in technical quadruplicates. Statistical analysis was performed with the unpaired *t*-test. *P < 0.05, **p < 0.01, and ****p < 0.001 compared to control condition. CTRL, control; PACs, proanthocyanidins-fraction; EGb, EGb 761^®^ extract.

Based on previous observations, we evaluated the impact of EGb 761^®^ and PACs on the bioenergetic profile of human neuroblastoma cells by performing the Seahorse XF Mito Stress Test. We observed a statistically notable increase in the OCR (EGb 761^®^ 10 μg/mL, *p* = 0.0017, PACs 1 μg/mL, *p* = 0.0041) (Figure 3A) and ECAR (EGb 761^®^ 10 μg/mL *p* = 0.0003, PACs 1 μg/mL, *p* = 0.0001) (Figure 3B) in both treatment groups. The amelioration of the extracellular acidification rate (ECAR) and oxygen consumption rate (OCR) as indicators of glycolysis and OXPHOS, respectively, indicate a metabolic shift in the cells toward a more energetic and metabolic state in comparison to the untreated control (Figure 3C). This is further illustrated by the energy phenotype map, where both EGb 761^®^ and PACs treatments shift cells from a quiescent phenotype into the energetic quadrant. Notably, PACs induced a slightly stronger enhancement in oxidative capacity (OCR) relative to ECAR, suggesting a more pronounced stimulation of mitochondrial function. Overall, both treatments promote a similar bioenergetic reprogramming in human neuroblastoma cells, favoring a more metabolically active phenotype.

### EGb 761^®^ and PACs promote neurite outgrowth and connectivity

**FIGURE 4:**
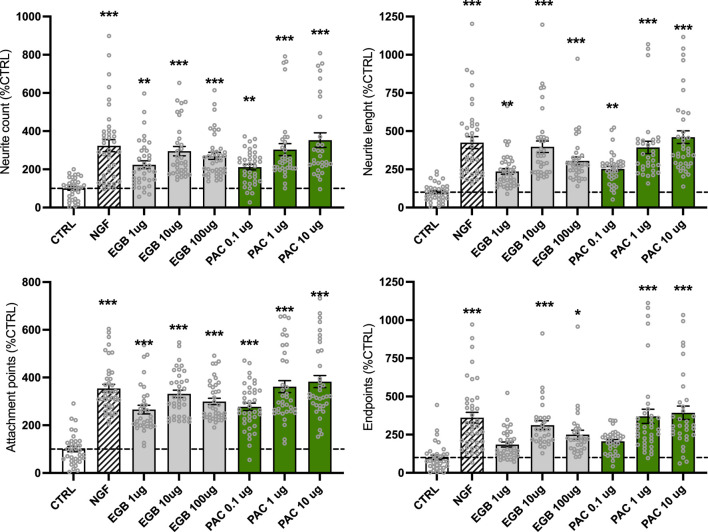
EGb 761^®^ and PACs increased the neurite outgrowth in the human neuroblastoma cells. In total, approximately 10,000 cells and 57,000–101,000 neurites were evaluated. Images were taken with the Cytation 3 cell imaging multi-mode reader and analyzed with the ImageJ neurophology software to evaluate parameters of neuroplasticity. Values represent the mean ± SEM of three independent experiments and were normalized to the cell count. Data are presented as % normalized to the CTRL group. Per condition, 3,400–11,300 cells were analyzed in total. Each open circle represents one image. One way ANOVA and *post hoc* Dunnett’s multiple comparisons versus CTRL. *P < 0.05, **p < 0.01, and ***p < 0.001. CTRL, control; PACs, proanthocyanidins-fraction; EGB, EGb 761^®^ extract.

Neuroplasticity is a process with high energy demand. As treatment with EGb 761^®^ and PACs can increase the energetic state of cells, it was of interest to study whether we can detect an increase in outgrowth measures. Imaging neurite outgrowth in human neuroblastoma SH-SY5Y cells (×20, Supplementary Figure S4) using the Cytation 3 cell imaging multi-mode reader allows for both visualization and quantification of neurite formation and their projections between cells (Figure 4). After 3 days of treatment with EGb 761^®^, concentrations of 10 μg/mL (+195%, *p* < 0.001) and 100 μg/mL (+170%, *p* < 0.001) were found to be the most effective, boosting the neurite count to nearly the same extent as NGF (+223%, *p* < 0.001) compared to that in untreated cells (CTRL). An amount of 1 μg/mL of EGb 761^®^ increased the neurite count by 124% (*p* = 0.002). Similarly, PACs treatment showed comparable effects and enhanced neurite count by 111% (*p* = 0.008), 203% (*p* < 0.001), and 253% (*p* < 0.001) for the 0.1, 1, and 10 μg/mL concentrations, respectively. In addition, treatment with 10 and 100 μg/mL of EGb 761^®^ significantly enhanced neurite length by up to 298% (*p* < 0.001) and 205% (*p* < 0.001), respectively. With an increase of 153% (*p* = 0.001), 293% (*p* < 0.001), and 360% (*p* < 0.001) for the 0.1, 1, and 10 μg/mL PACs treatments, respectively, PACs seem to have a stronger effect on neurite length. PACs treatment also significantly increased the number of attachment points, with 262% and 282% increases at 1 and 10 μg/mL, respectively (*p* < 0.001), exceeding the effects of both NGF (+254%, *p* < 0.001) and EGb 761^®^ (165%, 232%, and 199% increases at 1, 10, and 100 μg/mL, respectively; all *p* < 0.001). Similar trends were observed for endpoints, where PACs at 10 μg/mL increased counts by 292% (*p* < 0.001), surpassing both NGF (+261%) and EGb 761^®^ (up to 212%, *p* < 0.001).

## Discussion

Most research studies focus mainly on the effects of EGb 761^®^ in the treatment of hearing disorders, mild cognitive decline, and dementia including Alzheimer’s disease (DeFeudis, 2003; Benninghoff and Perneczky, 2022; Noor-E-Tabassum et al., 2022). However, only limited studies have directly examined the effects of EGb 761^®^ and its individual constituents—particularly PACs—on mitochondrial function. The underlying mechanisms of action remain insufficiently characterized, despite consistent findings that EGb 761^®^ shows antioxidant activity, scavenges free radicals, and supports mitochondrial function. Among its constituents, terpene trilactones have been implicated in mediating several of these effects. Yet, a substantial portion of the extract—approximately 70% of its mass—remains uncharacterized (European Medicines Agency, 2015). Given this and the observed variability in extract composition between manufacturers (Germer et al., 2024), further investigation into the pharmacological contribution of PACs and the other uncharacterized 70% portion of EGb 761^®^ is of substantial importance.

The antioxidant properties of EGb 761^®^ have been validated across a broad range of *in vivo* and *in vitro* studies (Abdel-Kader et al., 2007; Shi et al., 2010; Ude et al., 2013; Anaya-Fernández et al., 2024; Xia et al., 2024), underlining a higher radical scavenging potency of the flavonoid fraction than the terpenoid fraction (Ramassamy et al., 1993). As both PACs and flavonoids belong to the polyphenol class, their antioxidant function is expected (Cao et al., 2018). PACs from Ginkgo and other sources have demonstrated free radical scavenging capacity in cell-free systems (Qa’dan et al., 2011), in a neuronal cell line (Sens-Albert et al., 2021), in retinal pigment epithelial cells (Li et al., 2021), and in animal models (Cao et al., 2018).

The substantial antioxidant activity, surpassing even well-established vitamin C and E, can be attributed to the polyhydroxy phenolic nature of the substance class, which exerts its anti-oxidative function mainly by adjacent aromatic hydroxy groups, which can be strong proton and electron donors (Ramassamy et al., 1993; Bagchi et al., 2000; Santos-Buelga and Scalbert, 2000; Hatano et al., 2002; Beninger and Hosfield, 2003; Beecher, 2004; Ho et al., 2010). In our study, we successfully illustrated the ability of both EGb 761^®^ and PACs to scavenge mitochondrial superoxide. Both treatments yielded notable effects, with PACs exhibiting a marginally higher maximal effect level than EGb 761^®^, thus supporting their role in mediating the antioxidant properties of the extract (Abdel-Kader, 2009; Barbalho et al., 2022).

*Ginkgo biloba* has also been shown to improve cerebral energy metabolism, protecting against OXPHOS uncoupling and maintaining ATP levels by elevating the respiratory control ratio (DeFeudis and Drieu, 2000). It might also prevent hypoxia-induced ATP depletion *in vitro* and support glycolytic activity and glucose transport (Janssens et al., 1995). In line with these observations, our study demonstrated that both EGb 761^®^ and PACs increased ATP levels in SH-SY5Y cells after 24 h of treatment. This supports the idea that PACs, in addition to flavonoids and terpenes, contribute to EGb 761^®^ energy-modulating properties.

Our analysis of mitochondrial bioenergetics revealed a treatment-induced increase in both mitochondrial respiration (OCR) and glycolytic activity (ECAR), indicating a metabolic shift toward a more energetic phenotype. PACs induced a slightly greater increase in OCR than EGb 761^®^, suggesting a pronounced stimulation of mitochondrial oxidative metabolism. The observed enhancement in MMP further supports the link between PACs and improved ATP production *via* oxidative phosphorylation (DeFeudis and Drieu, 2000; Montes et al., 2015; Müller et al., 2019). Interestingly, EGb 761^®^ at the highest concentration tested (100 μg/mL) resulted in lower ATP levels, a finding that may indicate increased ATP consumption in downstream cellular processes, such as neuroplasticity or stress response mechanisms (Schindowski et al., 2001; Stoll et al., 2007; Lejri et al., 2019).

One of the most remarkable effects observed was the stimulation of neurite outgrowth by both EGb 761^®^ and PACs. Treatment with EGb 761^®^ increased the neurite count, length, and branching in a concentration-dependent manner, reaching levels comparable to those induced by NGF. PACs demonstrated comparable, and in some cases superior, effects on all neurite parameters, strongly supporting their role in promoting neuroplasticity. These findings align with the previous literature showing that EGb 761^®^ supports mitochondrial integrity and enhances neurite formation under oxidative stress conditions (Eckert, 2005; Abdel-Kader et al., 2007). Additionally, neurite-promoting effects have been linked to *Ginkgo biloba* extracts through the activation of pathways such as Akt/mTOR and Wnt/β- (Lejri et al., 2019; Li et al., 2018).

Our study identified key mitochondrial adaptations to EGb 761^®^ and PACs, including increased mitochondrial mass and significant upregulation of PPARGC1-α expression, a transcriptional coactivator in mitochondrial biogenesis and energy homeostasis. The coordinated increase in mitochondrial mass, metabolic activity, and PPARGC1-α expression points to a broader mechanism by which EGb 761^®^ and PACs improve mitochondrial function and cellular energy metabolism. The marked increase in metabolic activity observed in MTT assays supports the idea that both EGb 761^®^ and PACs enhance cellular energy availability. We hypothesize that treatment with both EGb 761^®^ and PACs initially led to elevated ATP levels, only to be subsequently utilized in vital cellular pathways and metabolic adaptations as neurite outgrowth requires a substantial amount of energy.

In conclusion, these findings illustrate that PACs are not merely passive components but active contributors to the biological effects of EGb 761^®^. Both compounds improved mitochondrial function, enhanced ATP production, promoted mitochondrial biogenesis, and stimulated neurite outgrowth *in vitro*. Among the main constituents of EGb 761^®^—including flavonol glycosides (∼20%), ginkgolides (∼3%), bilobalide (∼3%), and PACs (∼7%)—PACs clearly demonstrate neuroprotective and metabolic effects that complement those of other well-characterized components.

Ginkgolides promote neurite growth and protect against oxidative and ischemic damage *via* neurotrophic signaling (Ramassamy et al., 1993; Yang et al., 2018), whereas bilobalide enhances mitochondrial function, stabilizes membranes, reduces neuronal apoptosis (Zhang et al., 2022), and mitigates oxidative stress (Barth et al., 2021). Flavonol glycosides, particularly quercetin and kaempferol, act as antioxidants, reducing oxidative stress and enhancing neuroplasticity *via* CREB-BDNF pathway upregulation (Hou et al., 2010). These findings suggest synergistic interactions among PACs, flavonols, ginkgolides, and bilobalide within EGb 761^®^, with PACs showing comparable pharmacological activity. PACs should thus be recognized as essential bioactive components of EGb 761^®^. Further research is needed to elucidate the largely uncharacterized 70% portion of quantified Ginkgo extracts to better understand its contributions to EGb 761^®^’s therapeutic potential. In particular, advanced disease-relevant cell models are required to clarify the effects of EGb 761^®^ and PACs under conditions that more closely mimic the pathological state. As part of this effort, we aim to investigate the impact of EGb 761^®^ and PACs on iPSC-derived neurons (Lejri et al., 2024; Szabo et al., 2024) and induced neurons from fibroblasts (Varghese et al., 2025), focusing specifically on how cellular age influences the response to treatment.

## Data Availability

The raw data supporting the conclusions of this article will be made available by the authors, without undue reservation.
