# Supplementary material for: Proanthocyanidins from Ginkgo extract EGb 761® improve bioenergetics and stimulate neurite outgrowth in vitro
Source: Front Pharmacol. 2025 Jun 12;16:1495997. doi: 10.3389/fphar.2025.1495997 (PMC12198615; doi:10.3389/fphar.2025.1495997)
Supplement: Supplementary file 1 [file DataSheet1.zip › supplementary file/supplementary file table2 PACs in EGb761 Lejri et al 2025.pdf]

	Experiment	CTRL	EGB 1 ug/ml	EGB 10 ug/ml	EGB 100 ug/ml	PACS 0.1 ug/ml	PACS 1 ug/ml	PACS 10 ug/ml
Fig.2A MTT (abs @ 550 nm)	XP1	0.3633	0.4544	0.4926	0.4615	0.4402	0.4877	0.4417
	Std. Error of Mean	0.01599	0.01545	0.03568	0.005527	0.03178	0.032	0.03352
	XP2	0.364	0.4526	0.4631	0.5619	0.4215	0.4234	0.4313
	Std. Error of Mean	0.01107	0.01408	0.02491	0.01578	0.0312	0.01441	0.0274
	XP3	0.3749	0.4241	0.4411	0.4667	0.4293	0.4083	0.4649
	Std. Error of Mean	0.007222	0.0208	0.01886	0.02518	0.02049	0.01109	0.00748
	XP4	0.4684	0.4992	0.5228	0.5336	0.4792	0.4964	0.523
	Std. Error of Mean	0.01224	0.004865	0.02091	0.01607	0.03016	0.01427	0.02391
	XP5	0.4783	0.593	0.5765	0.5611	0.571	0.5092	0.6252
	Std. Error of Mean	0.006088	0.07791	0.09152	0.03213	0.03885	0.02632	0.02502
Fig.2B MitoMass (Fluorescence Intensity)	Experiment	CTRL	EGB 1 ug/ml	EGB 10 ug/ml	EGB 100 ug/ml	PACS 0.1 ug/ml	PACS 1 ug/ml	PACS 10 ug/ml
	XP1	0.1507	0.1608	0.1646	0.1642	0.1719	0.1888	0.2103
	Std. Error of Mean	0.002037	0.001944	0.003084	0.001131	0.001881	0.002746	0.001948
	XP2	0.2171	0.2354	0.2432	0.212	0.2334	0.2468	0.2307
	Std. Error of Mean	0.007801	0.002943	0.008218	0.004301	0.006831	0.008285	0.0105
	XP3	0.1785	0.1952	0.2015	0.2158	0.221	0.2197	0.2293
	Std. Error of Mean	0.005023	0.00307	0.003294	0.004949	0.005721	0.005264	0.004766
Fig.2C PGC1a (Fold Change GAPDH)	Experiment	CTRL	EGB 10 ug/ml	PACS 1 ug/ml				
	XP1	0.9468	1.67	1.213				
	Std. Error of Mean	0.1158	0.2771	0.1559				
	XP2	3.223	3.983	3.519				
	Std. Error of Mean	0.6665	0.9896	0.6165				
	XP3	0.7671	1.414	1.124				
	Std. Error of Mean	0.3534	0.162	0.01051				

Suppl. Table 2. The table presents the mean of raw values from each independent experiment included in Fig. 2, along with the corresponding standard error of the mean (SEM) for each dataset.
