# Supplementary material for: Proanthocyanidins from Ginkgo extract EGb 761® improve bioenergetics and stimulate neurite outgrowth in vitro
Source: Front Pharmacol. 2025 Jun 12;16:1495997. doi: 10.3389/fphar.2025.1495997 (PMC12198615; doi:10.3389/fphar.2025.1495997)
Supplement: Supplementary file 1 [file DataSheet1.zip › supplementary file/supplementary file table1 PACs in EGb761 Lejri et al 2025.pdf]

	Experiment	CTRL	EGB 1 ug/ml	EGB 10 ug/ml	EGB 100 ug/ml	PACS 0.1 ug/ml	PACS 1 ug/ml	PACS 10 ug/ml
Fig.1A MitoSOX (fluorescence intensity, arbitrary unit)	XP1	0.02686	0.02482	0.0251	0.02256	0.02355	0.02401	0.02358
	Std. Error of Mean	0.0004682	0.0003323	0.0006008	0.0004744	0.0007352	0.0005352	0.0003951
	XP2	0.03156	0.03238	0.03241	0.03185	0.03084	0.03241	0.03009
	Std. Error of Mean	0.001218	0.002297	0.002401	0.002432	0.00257	0.002586	0.002053
	XP3	0.02827	0.02598	0.02556	0.02523	0.02608	0.02579	0.02607
	Std. Error of Mean	0.0006342	0.0009916	0.001531	0.0007001	0.001952	0.0009631	0.003068
Fig.1B MMP (Fluorescence Intensity)	Experiment	CTRL	EGB 1 ug/ml	EGB 10 ug/ml	EGB 100 ug/ml	PACS 0.1 ug/ml	PACS 1 ug/ml	PACS 10 ug/ml
	XP1	1070	1220	1274	1266	1280	1419	1370
	Std. Error of Mean	12.91	59.7	59.2	70.87	23.16	65.36	62.86
	XP2	1988	2284	2856	2611	2727	2649	3100
	Std. Error of Mean	63.62	80.12	311.2	96.15	103.8	144.8	160.9
	XP3	1018	1284	1425	1369	1641	1757	1676
	Std. Error of Mean	23.55	31.11	81.04	47.39	82.19	84.99	73.1
	XP4	1141	1280	1367	1211	1513	1537	1414
	Std. Error of Mean	26.6	52.11	42.22	26.66	48.8	36.51	62.25
	XP5	1016	1134	1130	1171	1362	1441	1500
	Std. Error of Mean	24.15	39.28	22.6	28.69	20.94	36.76	71.31
Fig. 1C ATP (μM)	Experiment	CTRL	EGB 1 ug/ml	EGB 10 ug/ml	EGB 100 ug/ml	PACS 0.1 ug/ml	PACS 1 ug/ml	PACS 10 ug/ml
	XP1	1.05	1.107	1.109	1.07	1.075	1.116	1.102
	Std. Error of Mean	0.03848	0.006396	0.009597	0.01057	0.01443	0.004807	0.008345
	XP2	1.26	1.268	1.29	1.238	1.278	1.315	1.264
	Std. Error of Mean	0.01045	0.01075	0.008659	0.006986	0.00459	0.01917	0.01067
	XP3	1.014	1.034	1.032	1.013	1.019	1.022	1.023
	Std. Error of Mean	0.00826	0.01125	0.01054	0.008177	0.01143	0.007749	0.01017
	XP4	1.362	1.363	1.384	1.355	1.358	1.364	1.349
	Std. Error of Mean	0.0105	0.01129	0.01722	0.009454	0.01574	0.01152	0.005829
	XP5	1.078	1.067	1.081	1.039	1.058	1.064	1.085
	Std. Error of Mean	0.01578	0.006872	0.005783	0.003712	0.004516	0.004256	0.004123

Suppl. Table 1. The table presents the mean of raw values from each independent experiment included in Fig. 1, along with the corresponding standard error of the mean (SEM) for each dataset.
