# Supplementary material for: Proanthocyanidins from Ginkgo extract EGb 761® improve bioenergetics and stimulate neurite outgrowth in vitro
Source: Front Pharmacol. 2025 Jun 12;16:1495997. doi: 10.3389/fphar.2025.1495997 (PMC12198615; doi:10.3389/fphar.2025.1495997)
Supplement: Supplementary file 1 [file DataSheet1.zip › supplementary file/supplementary file fig1 PACs in EGb761 Lejri et al 2025.pdf]

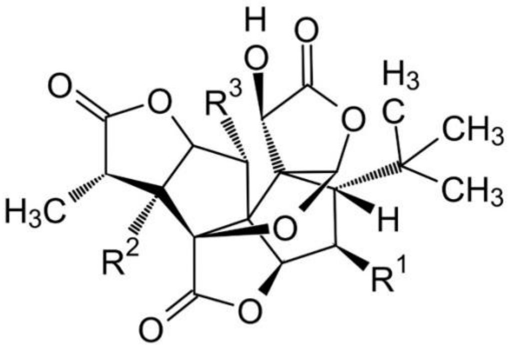

Ginkgolides A,B,C,J,M

Ginkgolide	R¹	R²	R³
**A**	-H	-OH	-H
**B**	-H	-OH	-OH
**C**	-OH	-OH	-OH
**J**	-OH	-OH	-H
**M**	-OH	-H	-OH

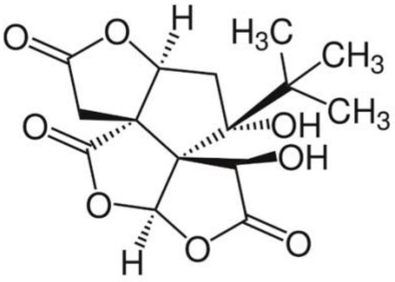

Bilobalide

Suppl.Figure 1. Chemical structure of the key terpene trilactones of EGb761®: Ginkgolides (upper panel) and bilobalide (lower panel). Terpenes represent one of the two principal categories of non-saponifiable lipids of plants. These molecules are made of units of the 5-carbon hydrocarbon isoprene. The particular terpenes that occur exclusively in Ginkgo biloba possess three lactone functions and a tertiary-butyl group.
