# Supplementary material for: Proanthocyanidins from Ginkgo extract EGb 761® improve bioenergetics and stimulate neurite outgrowth in vitro
Source: Front Pharmacol. 2025 Jun 12;16:1495997. doi: 10.3389/fphar.2025.1495997 (PMC12198615; doi:10.3389/fphar.2025.1495997)
Supplement: Supplementary file 1 [file DataSheet1.zip › supplementary file/supplementary file table 3 PACs in EGb761 Lejri et al 2025.pdf]

	Experiment	CTRL	EGB 10 ug/ml	PACS 1 ug/ml
Fig. 3A OCR	XP1	140.9	153.4	178.8
	Std. Error of Mean	6.869	3.543	4.996
	XP2	158.4	188.8	178
	Std. Error of Mean	9.003	4.443	2.043
	XP3	174.3	188.6	169.1
	Std. Error of Mean	2.868	2.561	5.403
	Experiment	CTRL	EGB 10 ug/ml	PACS 1 ug/ml
Fig.3B ECAR	XP1	51.45	42.97	46.55
	Std. Error of Mean	0.8508	1.837	0.9913
	XP2	43.28	68.1	67.05
	Std. Error of Mean	1.49	2.559	2.005
	XP3	34.27	52.2	54.69
	Std. Error of Mean	2.184	0.2872	1.1

Suppl. Table 3. The table presents the mean of raw values from each independent experiment included in Fig. 3, along with the corresponding standard error of the mean (SEM) for each dataset.
