# Supplementary material for: Proanthocyanidins from Ginkgo extract EGb 761® improve bioenergetics and stimulate neurite outgrowth in vitro
Source: Front Pharmacol. 2025 Jun 12;16:1495997. doi: 10.3389/fphar.2025.1495997 (PMC12198615; doi:10.3389/fphar.2025.1495997)
Supplement: Supplementary file 1 [file DataSheet1.zip › supplementary file/supplementary file fig4 PACs in EGb761 Lejri et al 2025.docx]

**
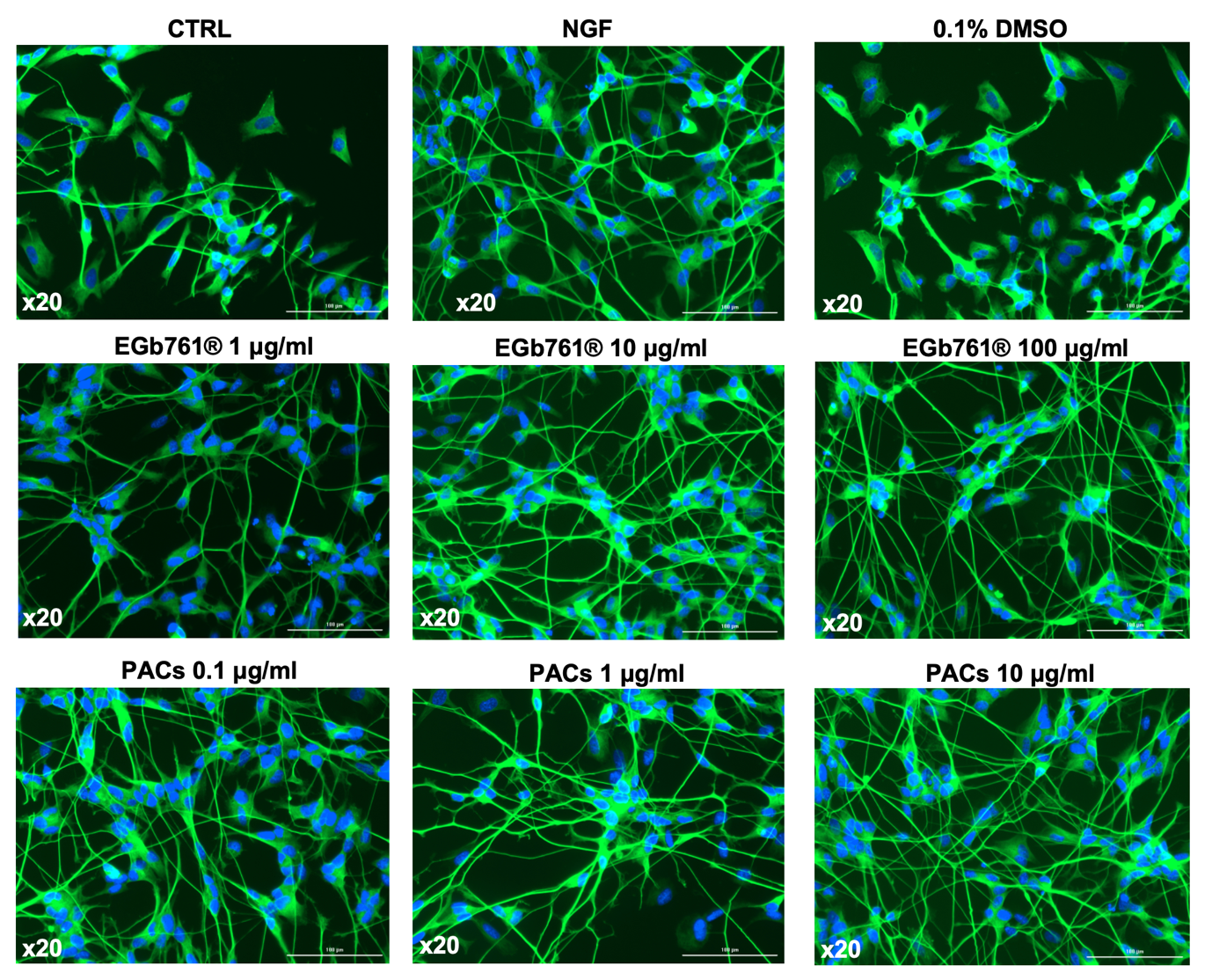
**

**Suppl. Figure 4. EGb 761® extract and PACs increased the formation of neurites in the human neuroblastoma cells.** Representative images of SH-SY5Y cells treated for 72 hours under various conditions: untreated control (CTRL), nerve growth factor (NGF), vehicle control (0.1% DMSO), EGb 761® at 1, 10, and 100 µg/ml, and PACs at 0.1, 1, and 10 µg/ml. Cells were stained for βIII-tubulin/Alexa488 (green) to visualize neurites, and DAPI (blue) to label nuclei. Images were captured using a Cytation 3 cell imaging multi-mode reader at 20x magnification. EGb 761® and PACs treatments resulted in increased neurite density and complexity compared to controls, with pronounced effects at higher concentrations. NGF-treated cells served as a positive control for neuritogenesis. Scale bars represent 100 µm.
