# Supplementary material for: Proanthocyanidins from Ginkgo extract EGb 761® improve bioenergetics and stimulate neurite outgrowth in vitro
Source: Front Pharmacol. 2025 Jun 12;16:1495997. doi: 10.3389/fphar.2025.1495997 (PMC12198615; doi:10.3389/fphar.2025.1495997)
Supplement: Supplementary file 1 [file DataSheet1.zip › supplementary file/supplementary file table 4 PACs in EGb761 Lejri et al 2025.docx]

**Suppl. Table 4. The table presents the mean of raw values from each independent experiment included in Fig. 4, along with the corresponding standard error of the mean (SEM) for each dataset.**
