# Supplementary material for: Proanthocyanidins from Ginkgo extract EGb 761® improve bioenergetics and stimulate neurite outgrowth in vitro
Source: Front Pharmacol. 2025 Jun 12;16:1495997. doi: 10.3389/fphar.2025.1495997 (PMC12198615; doi:10.3389/fphar.2025.1495997)
Supplement: Supplementary file 1 [file DataSheet1.zip › supplementary file/supplementary file fig2 PACs in EGb761 Lejri et al 2025.docx]

**
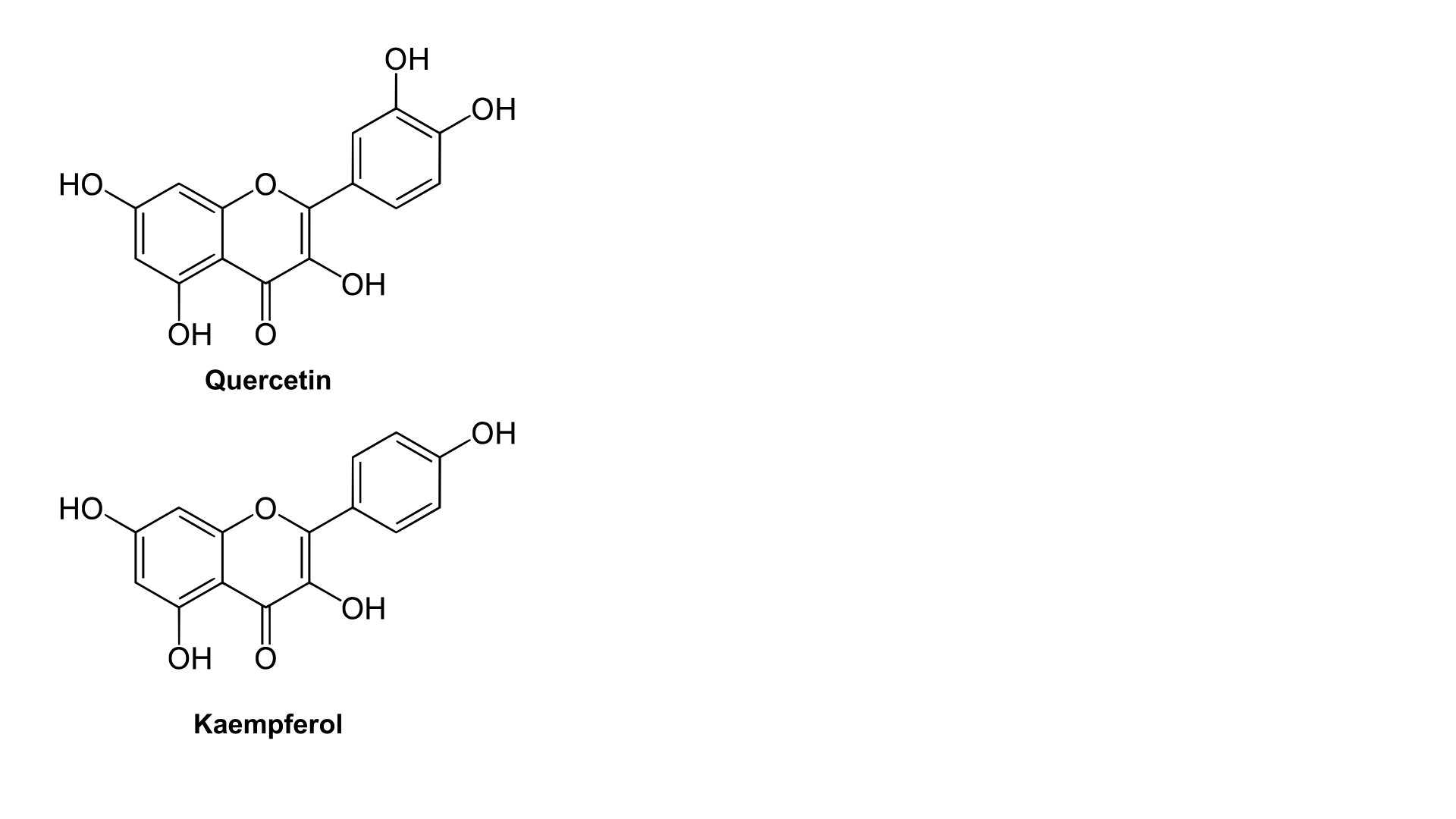
Suppl. Figure 2. Chemical structure of the key flavonols of EGb761®:** Quercetin (upper panel) and kaempferol (lower panel). Flavonols represent a subclass of flavonoids that have probably existed for more than a billion years and are widely distributed in the plant kingdom. The flavonoids present in EGb761® are primarily flavonol-O-glycosides of kaempferol and quercetin, and to a lower extent of isorhamnetin, i.e. combinations of the phenolic aglycon (quercetin, kaempferol) with sugars. Glucose and rhamnose are attached at various positions of the flavonol backbone.
