# Supplementary material for: Proanthocyanidins from Ginkgo extract EGb 761® improve bioenergetics and stimulate neurite outgrowth in vitro
Source: Front Pharmacol. 2025 Jun 12;16:1495997. doi: 10.3389/fphar.2025.1495997 (PMC12198615; doi:10.3389/fphar.2025.1495997)
Supplement: Supplementary file 1 [file DataSheet1.zip › supplementary file/supplementary file table 4 PACs in EGb761 Lejri et al 2025.pdf]

	Experiment	CTRL	NGF	EGB 1 ug/ml	EGB 10 ug/ml	EGB 100 ug/ml	PAC 0.1 ug/ml	PAC 1 ug/ml	PAC 10 ug/ml
Fig4. Neurite Count	XP1	805.1	2989	1804	2353	2442	1845	1930	2084
	Std. Error of Mean	157.1	325.9	251.3	293.5	311.5	164.5	92.6	216.2
	XP2	1261	2183	1791	2486	2485	1833	2257	2328
	Std. Error of Mean	97.59	257.4	149.8	106.7	158.7	161.8	188.8	261.6
	XP3	626.7	2625	2020	3227	2005	1998	3343	3889
	Std. Error of Mean	124.1	466.6	254.6	338.8	198.2	63.63	451.9	353.6
Fig.4 Neurite length (px)	Experiment	CTRL	NGF	EGB 1 ug/ml	EGB 10 ug/ml	EGB 100 ug/ml	PAC 0.1 ug/ml	PAC 1 ug/ml	PAC 10 ug/ml
	XP1	441.6	2029	906.9	1614	1640	1273	1232	1694
	Std. Error of Mean	96.13	226.4	101.2	201.6	355.5	133.1	78.37	177
	XP2	566.2	1709	1144	1486	1415	1066	2118	1934
	Std. Error of Mean	47.78	241.7	115.1	126.5	107.6	90.61	193.8	136.8
	XP3	340	1686	1111	2019	1093	1000	2225	2750
	Std. Error of Mean	63.03	335.2	156.4	278.9	118.6	28.39	382.2	348.2
Fig.4 Attachment points	Experiment	CTRL	NGF	EGB 1 ug/ml	EGB 10 ug/ml	EGB 100 ug/ml	PAC 0.1 ug/ml	PAC 1 ug/ml	PAC 10 ug/ml
	XP1	474.5	1675	1085	1500	1410	1274	1431	1525
	Std. Error of Mean	116.8	95.85	59.52	108.6	104.4	99.79	63.34	68.83
	XP2	559.1	1591	1157	1482	1311	1146	1343	1444
	Std. Error of Mean	49.73	78.35	69.95	69.19	30.24	77.09	85.94	95.14
	XP3	325.2	1427	1241	1378	1230	1254	1843	1905
	Std. Error of Mean	65.52	126.2	107.2	66.13	63.09	48.35	68.6	80
Fig.4 Endpoints	Experiment	CTRL	NGF	EGB 1 ug/ml	EGB 10 ug/ml	EGB 100 ug/ml	PAC 0.1 ug/ml	PAC 1 ug/ml	PAC 10 ug/ml
	XP1	483.4	1831	655.4	1570	1237	1067	1169	1439
	Std. Error of Mean	178	233.3	78.85	281.1	386.8	92.54	107.5	177.3
	XP2	466	1567	807.6	1313	1044	782.6	1115	1304
	Std. Error of Mean	32.28	257	96.55	138.4	104.2	82.41	113.6	162.9
	XP3	368.1	1345	940.8	1305	1110	929.6	2601	2632
	Std. Error of Mean	95.71	325.3	140.8	169	134.4	66.92	380	373.6

Suppl. Table 4. The table presents the mean of raw values from each independent experiment included in Fig. 4, along with the corresponding standard error of the mean (SEM) for each dataset.
