# Supplementary material for: Proanthocyanidins from Ginkgo extract EGb 761® improve bioenergetics and stimulate neurite outgrowth in vitro
Source: Front Pharmacol. 2025 Jun 12;16:1495997. doi: 10.3389/fphar.2025.1495997 (PMC12198615; doi:10.3389/fphar.2025.1495997)
Supplement: Supplementary file 1 [file DataSheet1.zip › supplementary file/supplementary file fig3 PACs in EGb761 Lejri et al 2025.pdf]

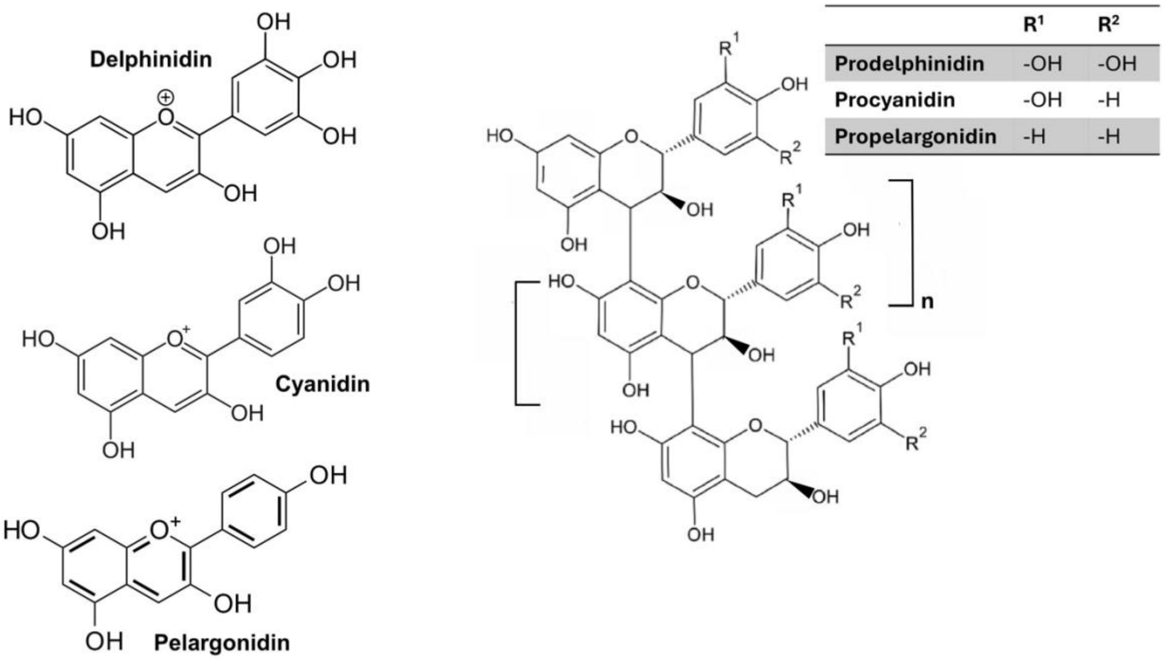

	R^1	R^2
**Prodelphinidin**	-OH	-OH
**Procyanidin**	-OH	-H
**Propelargonidin**	-H	-H

Suppl. Figure 3. Chemical structure of the key anthocyanidins and proanthocyanidins (PACs) of EGb761®: The left panel illustrates the anthocyanidins—delphinidin, cyanidin, and pelargonidin—which are flavonoid subclasses. The right panel displays their corresponding proanthocyanidins (PACs), such as prodelphinidins and procyanidins. PACs, also known as condensed tannins, are high-molecular-weight polyphenolic compounds formed by the polymerization of flavan-3-ols. Their characteristic structure includes two phenyl rings and one heterocyclic ring, with monomeric units linked through interflavonoid bonds, typically between C4 and C8 or C6. Based on their hydroxylation pattern and the anthocyanidins formed during acid-catalyzed depolymerization, PACs are sub-classified into procyanidins, propelargonidins, and prodelphinidins. In EGb 761®, PACs mainly consist of oligomeric to polymeric B-type dimers to hexamers, built predominantly from delphinidin and cyanidin, with minor contributions from pelargonidin.
